# A randomized, controlled, multicenter clinical study of the “improved sitting Wuqinxi” intervention for mechanically ventilated patients in the intensive care unit

**DOI:** 10.1097/MD.0000000000023898

**Published:** 2021-01-29

**Authors:** Guojin Xiao, Jing Liu, Li Zhang, Yan Yue, Xiangwen Weng, Zilin He, Lei Lv, Wendong Dong, Jing Li, Kunlan Long, Ren Yang

**Affiliations:** a The outpatient department; b Nursing Department; c Department of Critical Care Medicine, Hospital of Chengdu University of Traditional Chinese Medicine, Chengdu, China.

**Keywords:** clinical study, ICU, improved sitting Wuqinxi, mechanical ventilation, randomized controlled

## Abstract

**Introduction:**

Mechanical ventilation is a powerful tool for the treatment of various critical emergencies. However, surviving critically ill patients often have poor psychological and physiological outcomes. Prevention of various complications and adverse outcomes of mechanical ventilation is a priority and a challenge in the intensive care unit (ICU). Early intervention is the key to reducing complications and improving outcomes of mechanical ventilation in the ICU. As an auxiliary rehabilitation treatment, the improved sitting Wuqinxi intervention has recognized unique advantages. It has achieved beneficial therapeutic effects during early intervention in clinical practice. It can reduce the incidence of delirium, shorten the duration of mechanical ventilation, and prevent complications and secondary damages related to mechanical ventilation in the ICU. Therefore, the purpose of this study will be to explore the effect of improved sitting Wuqinxi on the clinical outcomes of mechanically ventilated ICU patients.

**Methods:**

This prospective, multicenter, randomized, single-blinded, parallel controlled clinical study will involve 160 patients who met the inclusion criteria. The patients will be randomly divided into the experimental and control groups. Both groups will be given standardized comprehensive western medicine treatment (including mechanical ventilation) and routine care in the ICU. Management of the experimental group will also include “improved sitting Wuqinxi,” with the treatment objective to observe the effect of the improved sitting Wuqinxi intervention on the clinical outcomes in mechanically ventilated ICU patients. The outcome measures will include the incidence of complications, duration of mechanical ventilation, length of ICU stay, and cost of hospitalization. In addition, the effect of the improved sitting Wuqinxi intervention on the safety indexes of mechanically ventilated ICU patients will be assessed and the clinical effects of the improved sitting Wuqinxi intervention will be comprehensively evaluated.

**Discussion:**

The purpose of this study will be to evaluate the effect of the improved sitting Wuqinxi intervention on the incidence of complications, duration of mechanical ventilation, length of ICU stay, cost of hospitalization, and safety indicators. If successful, it will provide a reliable, simple, and feasible auxiliary rehabilitation treatment scheme for mechanically ventilated ICU patients.

## Abstract 2

Supplemental Digital Content is available in the text

## 1 Introduction

With the continuous progress of the theory and technology of mechanical ventilation, its application in the intensive care unit (ICU) has become increasingly common. Patients who need long-term mechanical ventilation in the ICU account for 4% to 13% of the total number of patients.^[1,2]^ Mechanical ventilation has become a powerful tool for the treatment of acute or chronic respiratory failure and other diseases. Thus far, studies on mechanically ventilated patients have mainly focused on in-hospital mortality and short-term physiological endpoints.^[3,4]^ However, surviving critically ill patients often develop persistent long-term physiological and psychological complications.^[5]^

Ventilator dependence is a common phenomenon in mechanically ventilated ICU patients. Previous studies have shown that 39% mechanically ventilated patients have difficulty weaning from mechanical ventilation and that 18% patients have prolonged mechanical ventilation time.^[6]^ Long-term mechanical ventilation leads to various complications, such as ICU-acquired weakness (ICU-AW), dysfunction of the cardiac circulation system, metabolic disorders, and psychological disorders. ICU-AW is a common complication in mechanically ventilated patients. The incidence of ICU-AW can reach up to 33.0% to 82.0% in patients on mechanical ventilation for >4–7 days.^[7,8]^ Complications caused by mechanical ventilation not only prolong the duration of mechanical ventilation and length of ICU stay but also seriously damage patients’ functional recovery.^[9,10]^

Wuqinxi is a traditional Chinese sports healthcare method created by Hua Tuo, an ancient famous doctor. Wuqinxi is based on the theories of yin and yang, 5 elements, Zang Xiang, and meridians. Five birds (deer, ape, bear, bird, and tiger) belong to the 5 elements (wood, fire, soil, gold, and water), which also correspond to the 5 Zang organs (the liver, heart, spleen, lung, and kidney). Wuqinxi has recognized clear disease prevention and health care effects and can regulate the 5 Zang organs.^[11]^

Long-term bed rest and immobilization lead to serious complications in critically ill patients on mechanical ventilation. Early activity is a very safe and effective strategy for disease prevention and rehabilitation.^[12–14]^ Previous studies have shown the safety and feasibility of early activity in mechanically ventilated ICU patients.^[15–17]^ However, the current unified early activity plan is relatively simple and its clinical efficacy is unclear. The traditional sitting Baduanjin intervention can achieve good clinical effects in patients with sepsis-induced mechanical ventilation.^[18]^ Based on this finding and the clinical characteristics of ICU patients, the traditional sports health care skill Wuqinxi was adapted into the “improved sitting Wuqinxi” intervention for severe patients under the guidance of professional Wuqinxi practitioners. Wuqinxi can reduce the incidence of delirium in mechanically ventilated ICU patients, shorten the duration of mechanical ventilation, and prevent complications and damages secondarily to mechanical ventilation. However, sufficient clinical evidence is needed to confirm its effectiveness. Therefore, our goal of this study will be to evaluatethe efficacy and safety of the improved sitting Wuqinxi intervention in mechanically ventilated ICU patients using a prospective, randomized, controlled, and multicenter clinical study design.

## 2 Method and design

### 2.1 Design

**Figure 1:**
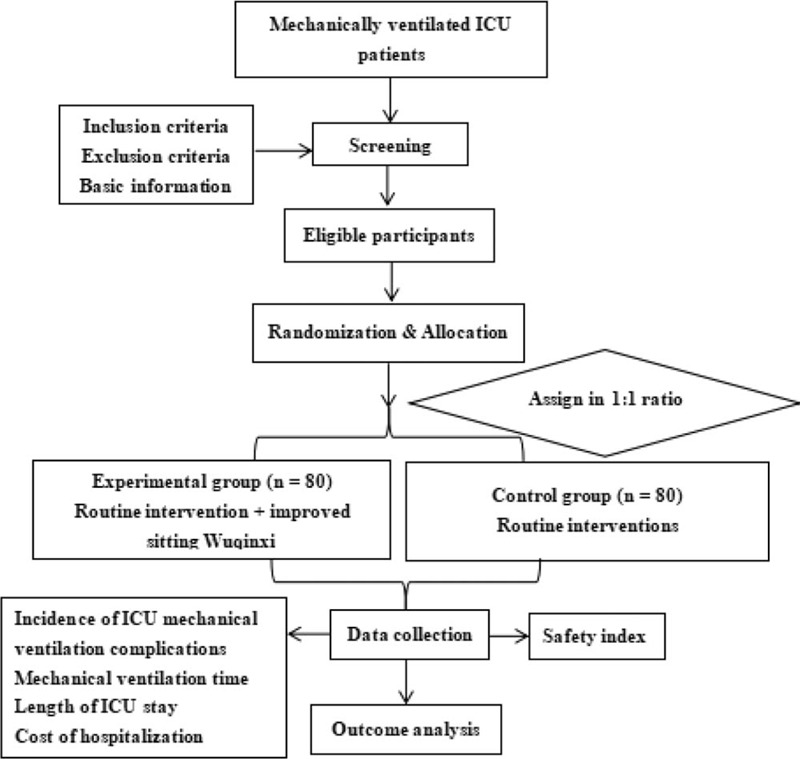
Flow chart of the study design. ICU = Intensive care unit.

**Figure 2:**
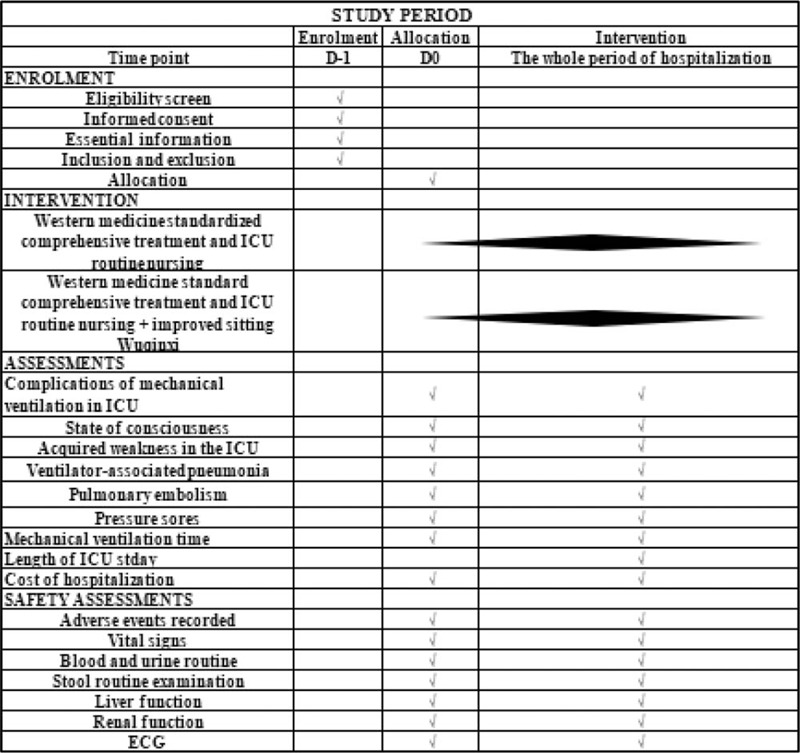
Schedule of enrollment, interventions, assessments, and data collection. Liver function index monitoring includes: ALT, AST, Tbil, ALP, GGT; Renal function index monitoring includes: BUN, Scr. ALP = Alkaline phosphatase, ALT = Alanine aminotransferase, AST = Aspartate aminotransferase, BUN = Blood urea nitrogen, ECG = electrocardiogram, GGT = γ-glutamyl-transferase, ICU = Intensive care unit, Scr = serum creatinine, Tbil = Total bilirubin.

The study will be designed as a prospective, multicenter, single-blinded, randomized, controlled clinical trial. Three medical institutions in China will be involved in the study—Affiliated Hospital of Chengdu University of Traditional Chinese Medicine (TCM), Affiliated Hospital of Chengdu University, and Leshan Hospital of TCM. The study has been registered with the China Clinical Trial Registration Center (No. ChiCTR2000033391, submitted on June 1, 2020, Version. V1.0). After obtaining written informed consent, 160 patients will be randomly divided into the experimental and control groups in a 1: 1 ratio. Both groups will be given standardized comprehensive treatment (including mechanical ventilation) and routine care in the ICU. The management of the experimental group will also include Wuqinxi in addition to the standardized treatment. The purpose of this study will be to assess the effect of the improved sitting Wuqinxi intervention in mechanically ventilated ICU patients. The impact of clinical outcomes will be reported in accordance with the statement of 2013 Standard Protocol Items: Recommendations for Interventional Trials (refer to Document 1 [http://links.lww.com/MD/D659] for the Standard Protocol Items: Recommendations for Interventional Trials list).^[19]^ The research flowchart is shown in Figure 1. The timing of the study and the baseline data collection plan are shown in Figure 2.

### 2.2 Moral certification

This study will be conducted in accordance with the Declaration of Helsinki (Edinburgh, 2000). The final revised draft (June 7, 2020 V1.0) and informed consent have been reviewed and approved by the Sichuan Regional Ethics Review Committee of The TCM/Medical Ethics Committee of The Affiliated Hospital of Chengdu University of TCM (No. 2019KL-031). All participants will also consent to the Standard Operation Training Procedure. If any amendments are made to the agreement, it will be reviewed and approved by the ethics committee again.

### 2.3 Recruitment

The patients will be recruited from Affiliated Hospital of Chengdu University of TCM, Affiliated Hospital of Chengdu University, and Leshan Hospital of TCM. Before the initiating the study, written informed consent signed by the participants or their immediate family members will be obtained to ensure that they understand the relevant information regarding the clinical study, including the research purpose, intervention measures, intervention time, and possible risks and benefits.

### 2.4 Sample size

According to the preliminary clinical research data, the total effective rate of “improved sitting Wuqinxi” in preventing and treating common complications and adverse outcomes in mechanically ventilated patients was statistically different between the groups. The test level was α = 0.05. The test efficiency was 1 - β = 0.80. The sample size was estimated using G∗Power (version 3.1.2, Franz faul, Universitat kiwl, Germany), and the effect size was set as d = 0.50. After calculation, the number of participants needed in each group was 64, totaling to 128 participants. Considering an attrition rate of ≤20%, the total number of participants required for this study was calculated to be 160, with 80 participants in each group.

### 2.5 Randomization and allocation concealment

Members of the Sichuan Traditional Chinese Medicine Evidence-Based Medicine center will use SAS 9.2 software (SAS, Cary, NC) to generate 160 random serial numbers. After screening and baseline evaluation, mechanically ventilated ICU patients will be randomly divided into the experimental and control groups in a 1:1 ratio. The group allocation number will be provided in a continuously numbered sealed envelope of carbon-free paper. The envelope will be kept by the research administrator, who will not be directly involved in the recruitment or follow-up of any participants, and their group allocation number will be disclosed later. The administrator will open the envelopes and provide participants with their group allocation number on the day of inclusion. The distribution of patients in each experimental center is shown in Table 1.

**Table 1:** Distribution of patients in each trial center.

Number of each center	Name of each center	Experimental group (patients)	Control group (patients)
01	Affiliated Hospital of Chengdu University of traditional Chinese Medicine	40	40
02	Leshan Hospital of traditional Chinese Medicine	20	20
03	Affiliated Hospital of Chengdu University	20	20
Total		80	80

### 2.6 Diagnostic criteria

The participants must meet the following diagnostic criteria: patients with chronic obstructive pulmonary disease (COPD), acute respiratory distress syndrome (ARDS), or postoperative respiratory failure requiring mechanical ventilation. For the diagnosis of COPD, the guidelines for diagnosis and treatment of COPD (2013 Revised Edition) formulated by the Chronic Obstructive Pulmonary Disease Group of the respiratory branch of the Chinese Medical Association will be followed (Table 2).^[20]^ The diagnosis of acute exacerbation of chronic obstructive pulmonary disease (AECOPD) is completely dependent on clinical manifestations, that is, patients complain of sudden changes in symptoms (baseline dyspnea, cough, and/or expectoration) beyond the daily variation range^[21]^; ARDS will be diagnosed according to the 2012 ARDS Berlin diagnostic criteria (Table 3).^[22]^ Postoperative respiratory failure mainly refers to respiratory failure after surgery for central nervous system diseases. The diagnostic criteria for respiratory failure will be in accordance with the Internal Medicine (Eighth Edition), edited by Junbo Ge and Yongjian Xu.^[23]^

**Table 2:** Diagnostic criteria for chronic obstructive pulmonary disease.

Clinical manifestation: Dyspnea, chronic cough, expectoration
Exposure history of risk factors: History of exposure to risk factors, such as smoking, occupational pollution, and biofuel exposure
Chest X-ray findings: Lung hyperinflation
Physical examination findings: Lung visual inspection: barrel chest; Auscultation: clear voice
Gold standard: Pulmonary function test: after inhalation of bronchodilator, FEV1/FVC < 70% was defined as persistent airflow limitation. COPD can be diagnosed after other diseases are excluded.

COPD = Chronic obstructive pulmonary disease, FEV1 = Forced expiratory volume in the first second, FVC = Forced vital capacity.

**Table 3:** Western medicine diagnostic criteria for acute respiratory distress syndrome.

Onset time: Clear risk factors within 1 week of onset or new/acutely aggravated respiratory symptoms within 1 week.
Causes of pulmonary edema: Respiratory failure cannot be explained completely by cardiac failure or fluid overload; if there is no relevant risk factor, objective examination (such as Doppler echocardiography) is needed to eliminate pulmonary edema with increased hydrostatic pressure.
Chest-X-ray: There is decreased opacity in both lungs, which cannot be explained by education, lobular atelectasis, or nodular shadows.
Oxygenation status:
Light: When CPAP or PEEP ≥5 cmH_2_O, 200 mmHg < PaO_2_/FiO_2_ ≤ 300 mmHg
Moderate: When CPAP or PEEP ≥5 cmH_2_O, 100 mmHg <PaO_2_/FiO_2_ ≤ 200 mmHg
Severe: When CPAP or PEEP ≥5 cmH_2_O, PaO_2_/FiO_2_ ≤100 mmHg

CPAP = continuous positive airway pressure, FiO_2_ = inhaled oxygen concentration, PaO_2_ = partial arterial oxygen pressure, PEEP = positive end-expiratory pressure; if the altitude is higher than 100 m, the following correction formula can be used: [PaO_2_/FiO_2_ × local atmospheric pressure/760]. In patients with mild ARDS, noninvasive CPAP can be used.

### 2.7 Qualification criteria

#### 2.7.1 Inclusion criteria

Only patients who meet the following criteria will be included:

(1) Age ≥18 years.
(2) Fulfillment of the diagnostic criteria of acute exacerbation of chronic obstructive pulmonary disease, acute respiratory distress syndrome, or postoperative respiratory failure requiring mechanical ventilation for ≥24 hours.
(3) Clear consciousness and the ability to act in accordance with instructions.
(4) Stable cardiopulmonary functions.
(5) Voluntarily participation after signing an informed consent.

#### 2.7.2 Exclusion criteria

The following patients will be excluded:

(1) Patients with rapidly progressive neuromuscular diseases
(2) Patients with Acute Physiology and Chronic Health Evaluation II score >30 and expected to die within 48 hours
(3) Patients with increased intracranial pressure, limb disability or loss, and unstable fracture
(4) Patients included in other clinical trials

### 2.8 Rejection, termination, and shedding criteria

All participants will be informed that they have the right to withdraw from the trial at any time and that they will receive standardized western medicine comprehensive treatment and ICU routine care regardless of participation. The reason for withdrawal will be recorded in their case report form (CRF).

All patients who do not meet the inclusion criteria will be excluded.

Patients who receive treatment but not in accordance to the regulations or those who have incomplete data will be excluded.

During the course of treatment, patients who withdraw voluntarily and those who experience adverse events (AEs) or serious AEs and not suitable for further treatment will be regarded as part of the attrition.

Patients with adverse reactions will be included in the statistics of adverse reactions. Patients who withdraw from more than 1/2 courses of treatment due to ineffectiveness will be included in the efficacy analysis.

## 3 Interventions

### 3.1 Treatment plan

#### 3.1.1 Control group

The control group will receive standardized comprehensive western medicine treatment (including mechanical ventilation) and routine care in the ICU.

#### 3.1.2 Experimental group

The experimental group will receive the same treatment as the control group and will additionally be treated with the improved sitting Wuqinxi intervention.

(1) Establish the standardized flowchart of “improved sitting Wuqinxi”for mechanically ventilated ICU patients
(2) There will be 3 groups of tiger opera at 09:00, 6 times in each group; 3 groups of bear play at 13:00, 6 times in each group; 3 groups of bird play at 15:00, 6 times in each group; and 3 groups of deer play at 19:00, 6 times in each group.
(3) Establish the risk assessment system

### 3.2 Observation index

#### 3.2.1 Incidence of ICU mechanical ventilation complications

The ICU consciousness disorder assessment form and delirium scale analysis system will be used to evaluate each patient state of consciousness. The presence of ICU-AW will be judged according to the British Medical Research Council grade 6. The incidence of ventilator-associated pneumonia, pulmonary infarction, and pressure ulcers will also be observed.

#### 3.2.2 Mechanical ventilation time

Data will be obtained to determine whether the mechanical ventilation time in ICU is shortened during the period from admission to the end of the trial.

#### 3.2.3 Length of ICU stay

The period from admission to discharge from the ICU will be recorded.

#### 3.2.4 Cost of hospitalization

Hospitalization expenses from admission to discharge from the ICU will be recorded.

### 3.3 Safety assessment

Vital signs; blood, urine, routine stool, liver, and kidney function test findings; and electrocardiogram will be recorded daily for the entire study duration. And any AEs observed will be documented in detail.

### 3.4 Compliance

The researchers will pay close attention to patients’ compliance and exclude patients with poor compliance. The researchers will also pay attention to the daily vital signs and relevant safety indicators of each patient and immediately assess whether they can continue to participate in the study if AEs related to the intervention measures or other disease conditions arise, and take effective intervention measures for any adverse reactions.

### 3.5 Adverse events

Any AE that occurs during the clinical study, whether or not it has a causal relationship with the experimental intervention, will be regarded as an AE. The AE report form will be completed. The occurrence time, severity, duration, measures, and results of AEs will be recorded. If any serious AEs occur, they will be reported to the local Adverse Drug Reaction testing center and ethics committee within 24 hours.

### 3.6 Data management and quality control

Before initiating the trial, all researchers will be required to attend a series of mandatory training courses to ensure that they fully understand the study protocol and treatment standards and ensure the accuracy and integrity of the clinical treatment and trial data recording. The research leader will monitor the progress of the study regularly. The data observer will first record all data in the paper version of the CRF and then double input the data into the electronic data acquisition system. The test supervisor will regularly check the progress of the study and the completeness and compliance of the CRF records. To ensure data objectivity, the data observers and statisticians will not know the group allocations of the patients. An independent quality inspector will monitor the entire study process.

### 3.7 Statistical analysis

Before analysis, the data of 2 participants with complete data will be carefully checked to ensure the accuracy of the data. All data will be analyzed by intention. SPSS version 22.0 (Chicago, IL) will be used to analyze the data. The measurement data will be represented as mean ± standard deviation. The paired t-test will be used to perform the before and after analyses of the same group. The chi-square test will be used for the counting data and the rank sum test will be used for grade data. The Kaplan–Meier method will be used for univariate analysis to explore each possible related factor. The multivariate Cox model will be used to adjust for variables with statistical significance (*P* < .05) in univariate analysis to detect the influence of these variables on the study measure outcomes.

### 3.8 Informed consent

We will provide each patient with a printed copy of the informed consent before initiating the trial, which will include the research name, research background, research methods, information on what the participants should do during the trial, inclusion/exclusion criteria, treatment plan and obligations, and possible adverse reactions caused by the intervention measures. We will make every effort to protect the privacy of patients’ personal medical data. Patients who voluntarily agree to participate in this study and those who will withdraw due to whatever reasons will continue to receive standardized western medicine comprehensive treatment and routine care in the ICU. Once the patients or their families sign the informed consent, their personal information and medical information will be officially used in this study.

### 3.9 Secrecy

Participants’ medical records will be kept in hospitals and will be accessible only to the researchers, research institutions, and ethics committees. No public report on the results of this study will reveal the personal identity of the participants. We will make every effort to protect the privacy of the participants’ personal medical data to the extent permitted by law. Personal and medical information will be kept confidential and the data will be stored in a safe and reliable place. Participants can request access to their personal information (such as their address and contact information) at any time and can modify this information, if necessary.

## 4 Discussion

Mechanical ventilation is usually used for respiratory support and treatment of respiratory failure in the ICU. Its primary goal is to improve gas exchange in the lung, reduce respiratory distress, promote the healing of the lung and respiratory tract, and reduce the risk of iatrogenic complications.^[24]^ However, ventilator-associated pneumonia, agitation,^[25]^ ICU-AW, cardiac dysfunction, and other complications, are significant problems that cannot be ignored by intensive care workers. Critically ill patients often have multiple organ dysfunctions, resulting in “five Zang organ disorders.” Modern research shows that Wuqinxi can regulate the 5 Zang organs. It has positive regulation and improvement effects on blood rheology, blood lipid levels, blood pressure, and other cardiovascular function indices; immune function indices such as peripheral blood T lymphocyte subsets and NK cells; as well as lung function, bone mineral density, muscle function, psychological state, and other functions.^[26,27]^ Through the practice of Wuqinxi, we can effectively delay aging, enhance various functions of the body, and improve disease resistance. In the early stage, we observed many benefits of the “improved sitting Wuqinxi” intervention in mechanically ventilated ICU patients. However, there is no multicenter, large sample, randomized controlled clinical study to further confirm its efficacy and safety. Therefore, it is necessary to conduct a prospective study to further the discussions. This study will have some limitations. First, due to the limitation of funds and conditions, the patients will not be followed up after discharge. Second, due to the implementation of the trial itself, the study will be a single-blinded trial; this may result in some bias in the observation of indicators. If the trial is successful, it will provide a reliable, simple, and feasible auxiliary rehabilitation treatment scheme for mechanically ventilated ICU patients.

## Acknowledgments

The authors are grateful to the Sichuan Science and Technology Program (http://202.61.89.120/) for funding this study. They also thank Editage (www.editage.cn) for English language edting.

## Author contributions

**Conceptualization:** Jing Liu, Li Zhang.

**Data curation:** Yan Yue, Xiangwen Weng, Zilin He, Jing Li.

**Methodology:** Guojin Xiao, Kunlan Long, Ren Yang.

**Supervision:** Guojin Xiao, Kunlan Long, Ren Yang, Lei Lv, Wendong Dong.

**Writing – original draft:** Guojin Xiao, Jing Liu, Li Zhang, Yan Yue.

**Writing – review & editing:** Kunlan Long, Ren Yang.

## Abbreviations

Abbreviations: AEs = adverse events, COPD = Chronic obstructive pulmonary disease, CRF = case report form, ICU = intensive care unit, ICU-AW = intensive care unit-acquired weakness, TCM = traditional Chinese medicine.
